# Impact of Veteran Status and Timing of PTSD Diagnosis on Criminal Justice Outcomes

**DOI:** 10.3390/healthcare6030080

**Published:** 2018-07-12

**Authors:** Brandt A. Smith

**Affiliations:** Department of Psychology, Columbus State University, Columbus, GA 31907, USA; smith_brandt@columbusstate.edu; Tel.: +1-706-565-1246

**Keywords:** PTSD, veterans, court, malingering

## Abstract

Previous research has demonstrated that jurors show a bias towards treatment for veterans with post-traumatic stress disorder (PTSD). The present research examines this bias when jurors are faced with cases of potential malingering, in which the defendant’s claim of PTSD is a perceived attempt to escape legal punishments. Trial vignettes, in which veteran status and PTSD diagnosis timing were manipulated, were used to explore this phenomenon. It was found that veterans who received their diagnosis after being arrested were found guilty more often, and were diverted to treatment less often, than those who were diagnosed before an arrest. This has critical implications for mental healthcare in that it is crucial to properly diagnose and treat people before they find themselves in court. Further, the negative outcomes in court demonstrate one of the severe social impacts of untreated or late-diagnosed PTSD.

## 1. Introduction

Post-traumatic stress disorder (PTSD) has received attention because of the prevalence of the condition in veterans from Operation Iraqi Freedom (OIF), Operation Enduring Freedom (OEF), and Operation New Dawn (OND). Associated with this is the supposed connection between some symptoms of PTSD and criminal behavior [1], for example, hypervigilance. The scientific evidence linking PTSD and criminal behavior is inconclusive [2], though still believed by some to resolved. Though in many cases defense attorneys avoid the so-called insanity-defense [3,4] PTSD has been shown to be a mitigating factor in criminal proceedings [5]. Unfortunately, there is a stigma associated with PTSD (real or perceived is immaterial) that could discourage people from seeking assistance and mental healthcare [6]. This stigma can lead to people not seeking help until they reach a negative social position and find themselves involved in the criminal justice system. A defense attorney may attempt to use PTSD as a defense for their client, which would require an evaluation by mental health professionals after the person has been arrested. This post-arrest diagnosis may be perceived as malingering by jurors and not serve to help a person who is suffering. This is the reason that defense attorneys often avoid the insanity defense, as it is often seen by juries to be an attempt to avoid responsibility [3,4]. For this reason, it is hypothesized that a post-arrest diagnosis is mistrusted and could be viewed as self-serving.

This phenomenon illustrates why it is necessary to diagnose and help those in need, before their lives are disrupted by criminal court proceedings. The present research examines one facet of this problem, PTSD diagnosis before or after an arrest. Previous research has shown a bias towards treatment for veterans with PTSD when verdict options beyond “guilty” and “not guilty” were available [7]. That research did not examine the influence of perceived malingering on juror verdicts. The present research was designed to remedy this. Examining the impact of said issues in a criminal court setting allows us to see the impact of perceived malingering of PTSD. This approach details, in a practical setting, the importance of early diagnosis and intervention to avoid future criminal justice issues and quality of life problems.

Jurors may view a post-arrest diagnosis of PTSD as an attempt to escape punishment, and some may note the ease by which PTSD can be malingered [8]. Some people may not seek treatment because of perceived stigma [9], which could leave them in a situation where their PTSD diagnosis is given after an arrest. This can lead to perceptions of malingering, which, in turn, would remove the bias towards treatment seen in previous research [7].

### 1.1. Malingering

Malingering and/or symptom exaggeration is fairly common in some instances, though, in criminal cases, researchers show a 19% prevalence [10]. Nearly one-fifth of criminal cases were found to have some evidence of malingering. This is problematic because a person could benefit from receiving a PTSD diagnosis if it helped them escape punishment. Whether or not the person truly has PTSD is immaterial to the purposes of the present research, as the examination is about juror responses to what could be malingered PTSD. The present research does not differentiate between exaggerated symptoms and malingering.

Research has shown that PTSD diagnoses are susceptible to malingering because the diagnosis relies heavily on a person’s self-report of symptoms [11]. There are several reasons why a person may malinger PTSD. Financial gains are obvious in that a person can receive money from their government in the form of disability payments. These payments can be substantial and result in other gains, money for school, and other considerations. Personal gain is particularly highlighted in the case of escaping criminal liability, where a PTSD diagnosis can alleviate personal responsibility. When there is a real or perceived gain from the diagnosis, it is more likely that a juror perceives the PTSD claim as malingered. No previous research has empirically examined the effects of perceived malingering in relation to juror verdicts.

### 1.2. Untreated PTSD

Taking into account the desire to avoid stigma associated with PTSD [6] and the notion that certain symptoms of PTSD can contribute to criminal behavior, a person can find themselves in court because of, at least in part, untreated PTSD. This reluctance to seek mental healthcare has been related to several other negative health outcomes. Untreated PTSD is persistent [12] and has been associated with damaging stress on family and social relationships [13]. Further, PTSD has been associated with general physical health symptoms [14,15], and it has a known comorbidity with alcoholism and other substance abuse disorders that carry their own health and social risks [16,17]. 

Veterans are disproportionately represented in the criminal justice system in the United States [18], in which veterans comprise approximately 9% of the prison population while comprising 7% of the overall population. This disproportionate representation of veterans in the criminal justice system is not accounted for by any social characteristics associated with veterans. The present research examined the impact of a post-arrest diagnosis on juror decision making. This was done to add to a small but growing corpus of knowledge related to the issue of PTSD and the criminal justice system. 

## 2. Materials and Methods 

The present study was a 2 (veteran status: veteran vs. non-veteran) by 3 (diagnosis timing: no diagnosis vs. post-arrest diagnosis vs. pre-arrest diagnosis) between-subjects design. The research protocol was approved by the university IRB (17-071). Two-hundred and twenty-eight people participated in this study. Two participants did not render a verdict, so they were excluded from analysis (final *N* = 226). The average age of the participants was 20.85 years (*SD* = 4.123; range 18–43 years old). The sample was predominately female (*n* = 166). The sample was comprised of college students in a small southeastern United States university.

Participants were randomly assigned to read a trial vignette that showed a defendant who was either a veteran or a non-veteran and revealed information about PTSD diagnosis. The trial vignette detailed a violent encounter in which a person suffered personal injury that was not life threatening. For the non-veteran condition, military service, or the lack thereof, was not mentioned. In the no diagnosis condition, there was no mention of PTSD. The no diagnosis condition was included in this study to serve as a control condition. After participants had read the trial vignette, they were asked to render a verdict of guilty, not guilty, or diverted to treatment. The participants that rendered a guilty verdict were presented with a short survey consisting of three items that measured the severity of the recommended punishment (“how long should the defendant spend in prison”). Participants who rendered a diverted to treatment verdict were shown a list of prohibitions and required activities that they could endorse for the defendant, and they were questioned as to how long the defendant should be under court supervision. All participants were given a short survey that assessed their trust of the criminal justice system. This was assessed to test for a potential moderator of verdict.

## 3. Results

Veteran status (non-veteran vs. veteran), timing of the diagnosis (no diagnosis vs. post-arrest diagnosis vs. pre-arrest diagnosis), and the interaction between the two variables were entered into a logistic regression analysis. The primary outcome—verdict—was given as guilty, not guilty, or diverted to treatment. Assumptions of linearity for independent variables and log odds were met. The model was significant, $\chi^{2}$ (10, *N* = 229) = 56.55, *p* < 0.0001, $R^{2}$ = 0.12. Veteran status did not predict verdict, $\chi^{2}$ (2, *N* = 229) = 0.995, *p* = 0.61. The timing of diagnosis predicted verdict, $\chi^{2}$ (4, *N* = 229) = 44.23, *p* < 0.0001, which shows that defendants who were diagnosed with PTSD after arrest are given guilty verdicts more often than defendants who were diagnosed before arrest. Both pre- and post-arrest diagnosis conditions were given fewer guilty verdicts than the no diagnosis condition (Table 1).

The interaction between veteran status and timing of diagnosis was a significant predictor of verdict, $\chi^{2}$ (4, *N* = 226) = 16.03, *p* = 0.003 (Table 2). Examining this interaction revealed that veteran status in the no diagnosis condition did not predict verdict, $\chi^{2}$ (2, *N* = 78) = 1.24, *p* = 0.54. Veteran status in the post-arrest diagnosis condition predicted verdict, $\chi^{2}$ (2, *N* = 77) = 6.41, *p* = 0.04, $r^{2}$ = 0.04. This shows that veterans with a pre-arrest diagnosis were found guilty less often than their non-veteran counterparts and were diverted to treatment more often. Veteran status in the pre-arrest diagnosis condition did predict verdict, $\chi^{2}$ (2, *N* = 74) = 8.93, *p* = 0.01, $r^{2}$ = 0.06, showing that non-veterans with a pre-arrest diagnosis of PTSD were given more guilty verdicts and less diversion to treatment. Unexpectedly, participant trust in the justice system did not affect verdict, all *p*-values > 0.28.

### 3.1. Guilty

Examining those participants that rendered a guilty verdict (*n* = 98) showed that veteran status did not predict severity of sentence, *F*(1, 97) = 0.884, *p* = 0.35. The timing of the diagnosis did, however, predict severity of the sentence, *F*(1, 97) = 6.319, *p* = 0.003, $r^{2}$ = 0.111. This shows that the non-diagnosis condition (*M* = 1.502, *SE* = 0.213) and the pre-arrest diagnosis condition (*M* = 1.738, *SE* = 0.262) did not differ, *t*(97) = −1.713, *p* = 0.76. Sentence severity for the pre-arrest diagnosis and the post-arrest diagnosis (*M* = 3.215, *SE* = 0.434) conditions, *t*(97) = 2.912, *p* = 0.013, differed. This shows the importance of early detection and diagnosis of PTSD, as it may serve to lessen sentences for people who become involved in the criminal justice system. The interaction between veteran status and timing of diagnosis was not predictive of sentence severity, *F*(2, 97) = 1.588, *p* = 0.21.

### 3.2. Diverted to Treatment

Examining those participants who rendered a diverted to treatment verdict (*n* = 71) did not differ on the amount of time that a person should be in supervised treatment regardless of veteran status, *F*(1, 70) = 0.071, *p* = 0.791, the timing of diagnosis, *F*(1, 70) = 0.814, *p* = 0.447, or the interaction between the two independent variables, *F*(2, 70) = 1.516, *p* = 0.227. However, there were differences for the restrictions of treatment by the experimental conditions (Table 3). To a great extent, veterans with a pre-arrest diagnosis of PTSD were held to a higher standard when diverted to treatment.

## 4. Discussion

The present research illustrated the need for early diagnosis of PTSD because of the manner in which diagnoses after the fact (post-arrest) could be viewed as malingering. Veterans who had been diagnosed with PTSD before they had been arrested were diverted to treatment more often than those who received a diagnosis after their arrest. Previous research has shown that jurors have a preference for diverting a veteran to treatment instead of finding them guilty [7]. The present research has built on those findings by including a condition in which a PTSD diagnosis could be perceived as a malingering or as an attempt to avoid responsibility in a criminal case.

Veterans may, because of the “hero” status that they have, be held to a higher standard than non-veterans. While sometimes good for the veteran—discounts with certain companies and other social benefits—the status can result in an expectation of better behavior. According to a study commissioned by the Chairman of the Joint Chiefs of Staff, veterans can deal with any challenges that they face [19]. This could explain why veterans appear to be held to a higher standard than non-veterans when diverted to treatment.

The impact of being diagnosed after there are criminal or social problems can be problematic for a defendant, in that PTSD, which could be seen as a mitigating factor, would not be considered such in a post-arrest diagnosis. Receiving a diagnosis of PTSD after an arrest could be seen as self-serving on the part of the defendant when that diagnosis is presented as a mitigating factor in the criminal case. This hypothesis was supported in the present research, which showed that pre-arrest diagnoses resulted in higher rates of being diverted to treatment.

People who are suffering may be less likely to receive proper treatment for their condition. Though the present research examined this problem for veterans, there is no reason to presume that the impact would not be the same for non-veterans. There are several barriers to treatment that minority groups face, such as finances or family/cultural inhibitions [20]. Another reason that there is a barrier to care is self-stigma. Self-stigma has been shown to be a barrier to care for US [21] and UK [22] armed forces veterans. This stigmatization of PTSD could create the situation in which a person would not be diagnosed until after an arrest. This post-arrest diagnosis could then be seen as self-serving.

In short, addressing PTSD early will provide more avenues for remedy. Late diagnoses, as seen in the present research, can result in negative consequences for people who are already suffering. Prison is far from an environment that would lend itself to proper mental healthcare [23]. Overcoming stigma, including self-stigma, and taking a proactive approach to diagnosing and caring for people who are suffering would lead to better outcomes for the individual and for society as a whole.

### Limitations and Future Directions

The present research is limited in that it used trial vignettes and a convenience sample of college students to test the hypotheses. This does call into question, to some degree, the generalizability of the findings. Previous research used similar methods [7]. Research comparing college samples and community samples have produced mixed results in that a community sample was more punitive than a college sample in some research [24], while other research has detected no differences between college and community samples [25]. Additional research is needed to determine if there is a difference for college and community samples when it comes to questions of PTSD and the criminal justice system.

Future research, using a community sample, should include information such as mock juror occupation, income, and other potential moderators. This information could useful for attorneys in the voir dire procedure. As the present research did not differentiate between exaggerated symptoms and malingering, future research should examine the impact of how juries understand this difference and integrate that understanding into their verdicts.

## Figures and Tables

**Table 1:** Verdict by diagnosis condition.

Diagnosis	Guilty	Not Guilty	Diverted
No Diagnosis	51	9	17
Pre-Arrest	17	16	43
Post-Arrest	36	21	16

**Table 2:** Verdict by interaction between diagnosis and veteran status.

Diagnosis	Veteran Status	Guilty	Not Guilty	Diverted
No Diagnosis	Non-Veteran	24	6	9
Post-Arrest	Non-Veteran	15	8	13
Pre-Arrest	Non-Veteran	13	7	18
No Diagnosis	Veteran	27	3	8
Post-Arrest	Veteran	21	13	3
Pre-Arrest	Veteran	4	9	25

**Table 3:** Treatment restrictions by condition.

Restriction	Non-Veteran/No PTSD	Non-Veteran/Post Arrest	Non-Veteran/Pre-Arrest	Veteran/No PTSD	Veteran/Post-Arrest	Veteran/Pre-Arrest
Alcohol Testing	8	10	12	8	2	16
Drug Testing	5	8	10	4	2	14
Anger Management	8	11	12	7	2	19
Therapy	5	10	18	5	3	20
Group Therapy	5	8	11	3	1	13
Prison: Failure to Complete	4	6	6	6	0	7
